# Delightful Traveling

**Published:** 1877-08

**Authors:** 


DELIGHTFUL TRAVELING.—This is the season for well-nigh universal travel, and the trains and boats come and go, fairly groaning with happy voyagers. But, if everybody felt as we do, the pleasure traffic over long lines of railroad would be small indeed. We go to Boston occasionally, and as often as we go by rail, we have occasion to regret it. It is one hurly-burly of dust, and din and smoke and jolt. Not all the water of the Cochituate can wash us clean after such an experience. We long ago learned that the journey eastward could be made one of perfect delight, by taking what is popularly known as the Fall River route. The finest steamers plying in our waters run on this line. We saw nothing in Europe at all comparable to the magnificent "Bristol," and the equally magnificent "Providence." Here luxuries abound. These boats are really splendidly-appointed floating hotels. You leave New York at five o'clock in the afternoon, and swiftly sail out into the sweet, pure, invigorating air of Long Island Sound. Health, strength and appetite and good spirits, come to the sweltered, debilitated citizen in every breath. In the morning you land at Newport, if that charming watering-place chances to be your haven of rest, or you take your choice of the five early trains which leave Fall River for Boston. The cost of the journey in money is less than by rail,.while if you compare the bodily wear and tear arising from eight or ten hours on the cars with the store of new life imparted by the peaceful night on the Sound, you will decide that the positive advantages of the Fall River line are worth ten times more than they cost. You will regret that the trip does not take days instead of hours.

